# Measuring Family Quality of Life: Scoping Review of the Available Scales and Future Directions

**DOI:** 10.3390/ijerph192315473

**Published:** 2022-11-22

**Authors:** Ghaleb H. Alnahdi, Arwa Alwadei, Flora Woltran, Susanne Schwab

**Affiliations:** 1 Special Education Department, College of Education, Prince Sattam Bin Abdulaziz University, Al-Kharj 11942, Saudi Arabia; 2 Centre for Teacher Education, Department of Education, University of Vienna, 1010 Vienna, Austria; 3 Optentia Research Focus Area, North-West University Vanderbijlpark, 1174 Hendrick Van Eck Boulevard, Vanderbijlpark 1900, South Africa

**Keywords:** family quality of life, intellectual disabilities, measurement instruments, scoping review

## Abstract

The lack of quality of life is a key issue for families with children with an intellectual disability. While the quality of life for people with disabilities has previously been researched as an individual variable, this has now shifted to include family members. The purpose of this study was to conduct a review of the studies measuring the quality of life of families with an intellectually disabled member, in order to identify the most commonly used scales and their psychometric properties. Method: Data were collected from six databases (ERIC, Web of Science, Scopus, CINAHL, MedLine, and Google Scholar), and this search yielded 3948 studies. One hundred and twenty studies that met the inclusion criteria were included in this study. Results: Nine scales were used in the last years to measure the quality of life for families with individuals with an intellectual disability. The Beach Center scale was the most common scale, followed by the Family Quality of Life Survey and the World Health Organization’s quality of life assessment (WHOQoL-BREF). The results showed that the included studies in the review lack the consideration of a broader population representing the different types of cultures with different socioeconomic backgrounds. Key aspects used to assess the FQoL are environmental factors (proximal and distal factors), as well as economic factors. Conclusion: Although the operationalization of the FQoL often incudes several subthemes, a general agreement regarding which domains of the FQoL need to be included in the measurements, and these do not exist right now. Moreover, multidimensional scales are still rare.

## 1. Introduction

For several years, the focus of the studies examining the quality of life of people with disabilities has been shifting from considering only the individual to including family [1]. This can be attributed to the movements for deinstitutionalization in the 1960s [2], and to the emergence of the inclusive approaches that aim to improve the environment, rather than the individual [3], and in which research is conducted with, rather than about participants [4]. As a result, family interaction and family relationships have recently been emphasized [5] and considered when planning disability intervention programs [6].

### 1.1. Defining the Family Quality of Life (FQoL)

There have been several attempts by researchers to define the family quality of life (FQoL). For instance, Park et al. [7] concluded that the FQoL is assured when “conditions where the family’s needs are met, and family members enjoy their life together as a family and have the chance to do things which are important to them” (p. 368). Furthermore, [8] describe the FQoL as the continuous interaction and communication between groups of individuals connected by a social system unit. Another rather recent definition, by Zuna et al. [9], considers the FQoL as “a dynamic sense of well-being of the family, collectively and subjectively defined and informed by its members, in which individual and family-level needs interact” (p. 262). Bhopti et al. [10], referring to the definition of the FQoL introduced by Zuna et al. [9], summarized that it can be understood as a collective, “because it is concerned with how the family members feel about their family’s quality of life, as a group” (p. 2). Furthermore, according to Bhopti et al. [10], the FQoL has a dynamic character, “because it can change in response to significant events, such as moving homes, the loss of a family member, or having a child with a disability” (p. 2).

#### Conceptualizing and Measuring the Family Quality of Life (FQoL)

Researchers around the world started to investigate the FQoL of individuals with disabilities, about 20 years ago [11], with most studies concentrating on intellectual disabilities (IDs) [1]. In 2003, Poston et al. [12] identified four functioning domains of the FQoL: (a) daily family life, (b) parenting, (c) family interactions, and (d) financial well-being. Over time, other scholars added several further components to the FQoL, such as relationships among family members, overall family well-being e.g., Hoffman et al. [13], and the well-being of individual family members e.g., Jansen-van Vuuren et al. [14]. Today, the FQoL is considered a multidimensional construct [6] that encompasses a variety of domains in family life [15] and is therefore relatively complex [6,16,17]. According to a review by Samuel et al. [6], numerous FQoL conceptualizations share that they are based on the individual’s QoL, by examining the domains as subscales and then grouping them, to explain the FQoL. However, the architecture of the FQoL scales uses individual domains and does not represent the overall concept of the FQoL [18,19]. Accordingly, the FQoL refers to the individual’s quality of life in the context of the family as a whole [18].

To date, numerous researchers from different countries have collaborated to develop measures of the specific domains of family life, such as family leisure e.g., Mactavish and Schleien [20], or the burden of family caregivers e.g., Phelps et al. [21]. Recently, however, scholars have recognized that a holistic approach that takes into account a variety of dimensions of family life, is necessary to understand the level of the quality of life experienced by families with children with an ID [6]. Furthermore, measuring various family processes and the relevant proximal and distal factors is critical to better understanding the families’ needs for services and support, in order to achieve positive outcomes for all stakeholders [6,22]. Despite previous efforts to develop new research instruments, current studies that focus on measuring the quality of life of families with children with an ID, using a multidimensional approach, often draw on one of the following two measurement scales [11,23]: The (1) *Beach Center Family Quality of Life Scale* [24] and the (2) *Family Quality of Life Survey-2006* [25]. Both scales differ greatly in the domains considered. Accordingly, the 2005 version of the *Beach Center Family Quality of Life Scale* includes five domains, namely (a) family interaction, (b) parenting, (c) emotional well-being, (d) physical and material well-being, and (e) disability support. However, the *Family Quality of Life Survey-2006* developed by Brown et al. [25] is somewhat more comprehensive. Thus, it incorporates the following nine components: (a) the health of the family, (b) financial well-being, (c) family relationships, (d) support from other people, (e) support from disability-related services, (f) influence of values, (g) careers and preparing for careers of family members, (h) leisure and recreation activities, and (i) community involvement. Both scales account for numerous within- and outside-family processes and ecological contexts [26], and have been tested in numerous surveys, e.g., [13,27,28]. Although they share some commonalities, such as considering all age groups, recognizing the family environment and assessing the effectiveness of family support systems, some differences can be identified [6,29]. Accordingly, both scales build on the divergent approaches to the operational definitions of the FQoL and capture a different number of dimensions [29]. Moreover, the *Beach Center Family Quality of Life Scale* was originally developed to measure the quality of life of U.S. families with children with disabilities, while the *Family Quality of Life Survey-2006* [25] is aimed at an international application [6]. Finally, Samuel et al. [6] summarized in their systematic review, that the *Family Quality of Life Survey-2006* [25] is better suited for planning and assessing support for families with children with disabilities, due to its scope and inclusion of multiple domains.

As shown above, there are currently few multidimensional scales that are most widely recognized and that have been used internationally to measure the QoL of families with children with an ID through quantitative approaches [6,13,18,19]. However, it is important to consider all of the members of the family, when delivering the support services to families of children with an ID [10].

There are few previous reviews that address the scales used in studies measuring the quality of life for families of children with intellectual disabilities e.g., [6,10,30]. To the best of our knowledge, no review of previous and current international studies using multidimensional scales to quantitatively measure the QoL of families of children with intellectual disabilities, has been published since 2016. Therefore, the current study may provide important insights that have implications for future projects aimed at providing quality support to the families of children with intellectual disabilities.

### 1.2. Aim of the Current Study

The current study is considered a scoping review of the international literature regarding the measurement of the family quality of life (FQoL). The review is guided by the following three research questions: (1) Which scales are most commonly used in studies about the FQoL? (2) What are the characteristic themes of the FQoL? (3) What are the psychometric properties of the scales used in studies about the FQoL?

## 2. Methodology

### 2.1. Literature Search

A scoping review methodology was chosen to provide an overview of the QoL scales for families of children with an ID because it offers the opportunity to provide a comprehensive synthesis of the findings on broader topics across a range of study designs [31]. The data collection followed the guidelines of the PRISMA-ScR [32]. Accordingly, we searched a total of six databases: ERIC, Web of Science, Scopus, CINAHL, MedLine, and Google Scholar. The search contained the following keywords in the title and/or abstract: “family quality of life” AND “intellectual disability”. It was also searched for “quality of life” OR quality AND “family” OR “caregivers” OR “parents” AND “intellectual disability” OR “cognitive disability” OR “disability”. The final searches were conducted in March 2022. Duplicates have been removed and no limit has been placed as to the date of publication.

### 2.2. Inclusion Criteria

Four main criteria were used to include studies in this review: first, the focus should be on the FQoL. Second, the study sample should include families of children with an ID. Third, it should use a scale to calculate the quantitative data. Fourth, it should be published in a peer reviewed paper. Finally, it should be written in English.

### 2.3. Exclusion Criteria

**Figure 1:**
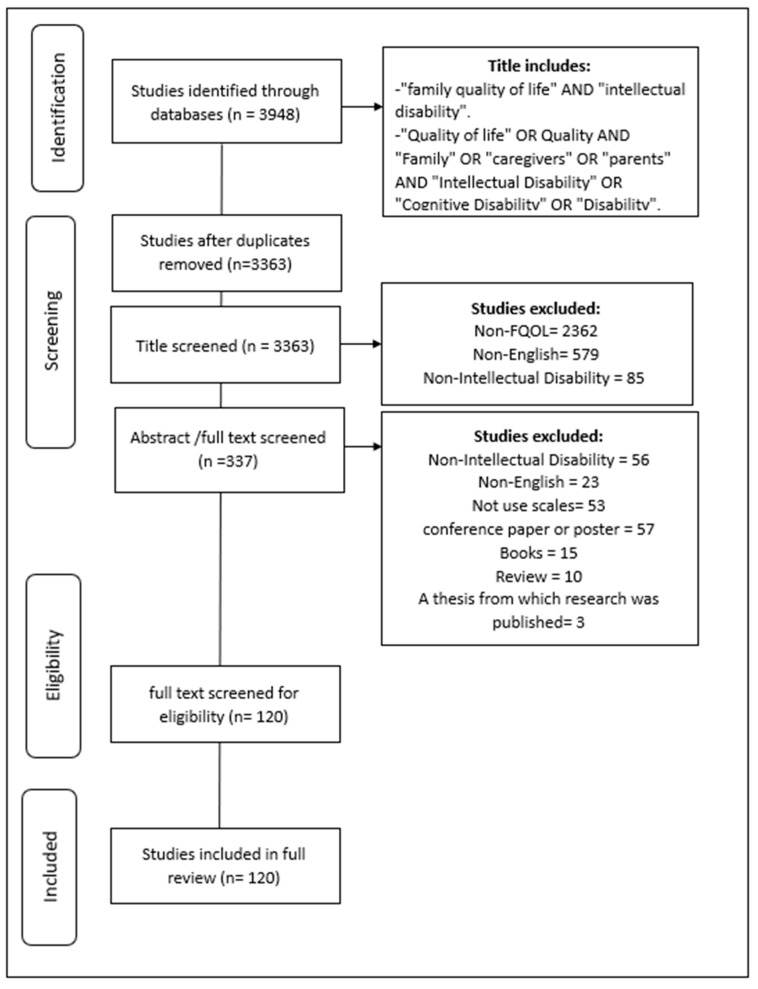
Steps of the data extraction.

Following a thorough search of the six databases, 3948 studies were identified as potentially relevant to this study. Following a title screen, 585 studies were removed due to duplication and 2362 studies were excluded because they addressed other topics unrelated to FQoL. In addition, the title screening excluded 85 studies because they were unrelated to intellectual disability and 579 because they were not in English, representing around 19% of the excluded studies at this stage. Following an abstract/full text screening of 337 studies, 120 studies were finally included in this study (see Figure 1 for the description of all exclusion criteria in each step).

### 2.4. Data Extraction

One researcher narrowed down the records obtained by searching the databases and excluded those that did not meet the inclusion criteria. The remaining records were reviewed by both researchers to reach an agreement on the inclusion and exclusion (Figure 1). The data were then extracted, identifying for each study, the type of scale used, the number of items, and the details of the domains covered by the study. In addition, further details on the samples and countries were obtained.

## 3. Results

### 3.1. Characteristics of the Studies Included in the Review

**Figure 2:**
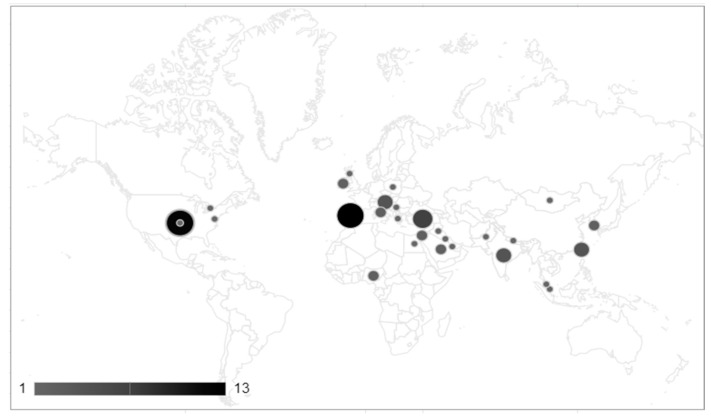
Map representing the samples from the different countries in the reviewed studies.

The scales used in the 120 studies are tabulated and summarized in Table 1. There are nine scales that were used in the studies that examined the FQoL for IDs in different cultures and countries, including four for an individual’s QoL used with a family member. Figure 2 shows that the samples included in the studies examined were located in several parts of the world. Therefore, the studies included in the current review lack the consideration of a broader population representing different types of cultures with different socioeconomic backgrounds. Most studies were conducted in the United States (*n* = 15), Spain (*n* = 13), Canada (*n* = 10), India (*n* = 9), Australia, and Turkey (*n* = 7). Africa as a continent, as well as South America, were the least representative continents in these samples. In the included studies, the scales were applied to multiple participants: families with IDs (*n* = 48), parents (*n* = 36), mothers (*n* = 9), siblings (*n* = 2), and caregivers (*n* = 25).

### 3.2. Scales Used in the Studies

The *Beach Center’s BC-FQoL scale* was the most commonly used scale. Forty-nine percent of all studies considered in the current study used the BC-FQoL and it was used in 27 countries. It consists of 25 items in five FQoL subdomains (family interaction, education, emotional well-being, physical/material well-being, and disability-related support). The BC-FQoL scale has been translated for use in several countries and tested for its psychometric properties. The domains are treated as subscales, as required by the aim of the study [40,41]. Some items were omitted due to their incompatibility with the study sample. For instance, item number twenty for the Korean sample [42] and the item “*My family receives dental care when needed*” because the sample belongs to the low-income group [41], as well as the phrase “*Support from school and workplace as children stay at home*” [43].

The *FQoLS-2006 Family Quality of Life Survey* was the second most commonly used FQoL scale in the studies considered, with a score of 25%, and it was used in 14 countries. It consists of two sections: Section A contains general questions, and Section B includes 54 items in nine domains (health, financial well-being, family relationships, support from others, support from services, influence of values, career, leisure, and community interaction). In addition, the FQoLS-2006 scale includes a dimension related to the overall QoL with an open-ended question, which contributes to a thorough understanding of the families’ perceived quality of life. The meaning and stability dimension was excluded because it did not fit the domain measurements [44]. In an adapted version used in an Australian study [45], the dimensions of support from others were divided into two dimensions: ‘practical support and emotional support’. In the Slovenian version of the scale [46], only six of the domains were included (health, financial well-being, support from others, support from disability-related services, interaction with the community, and overall family quality of life).

The *World Health Organization’s quality of life assessment* (*WHOQoL-BREF)* is the third most widely used instrument in the studies that were considered in the current review. Sixteen percent of the considered studies used it and it was used in 11 countries. It has been used with family members, such as mothers, fathers, siblings, and family caregivers of children and adults. The scale includes 24 items belonging to four dimensions: physical health, psychological well-being, social relationships and environment, and a facet on the overall QoL and general health. In the version that was used in Turkey, an additional dimension related to the Turkish environment was added [47]. In Iraq, the long version of the *WHOQoL-100* was used, which excludes the environmental dimension and contains 48 items [48].

In the studies conducted in Spain, two versions of the *CdVF-E* [34] scale were used. One version that is applied to persons who have reached the age of 18 and their families, considering 67 items, and another version that is applied to persons under the age of 18 and their families, considering 61 items. These items are distributed across seven domains: emotional well-being, family interaction, health, financial well-being, parental organization and skills, family accommodation, social integration, and participation. The other instrument commonly used in the Spanish studies, considered in the current review, is the *Families in Early Intervention QoL scale* [33]. It consists of 40 items distributed in three domains (family relationships, access to information and services, and child performance). In a study [49], another domain “general living situation” was added to the measures of the families’ perceptions of child achievement as an important influence on the FQoL [50].

The least used scales are the Family Quality of Life questionnaire Chinese FQoL-Q [34], the Quality of Life in Autism questionnaire (QoLA) [37], the Comprehensive Quality of Life scale (ComQoL) [39] and the Personal Wellbeing Index–Adult (PWI-A) [38]. The Family Quality of Life questionnaire Chinese FQoL-Q was first created in a study to develop a Chinese culturally specific FQoL scale with 35 items in seven domains: economy and leisure, physical and mental health, parenting, family communication, support from others, occupational support, and career development. In the Quality of Life in Autism questionnaire (QoLA) [37], 28 items are used to examine the role of the social support on the QoL of parents of children with an ID. The Comprehensive Quality of Life scale (ComQoL) assesses the QoL using an objective and a subjective subscale and includes seven domains: material well-being, health, productivity, intimacy, safety, place in community, and emotional well-being. In addition, the scale consists of two parts, the objective and the subjective components. Each dimension in the objective component includes three indicators for scoring. The subjective component is measured by determining the level of satisfaction in each dimension, weighted according to the importance perceived by the person. The Personal Wellbeing Index–Adult (PWI-A) was developed, based on the former ComQol scale [38] in order to measure the QoL of family caregivers. It consists of seven domains: standard of living, health, success in life, personal relationships, safety, community involvement, and future security with the optional addition of spirituality and religiosity.

### 3.3. Domains Included in the Current Scales

**Figure 3:**
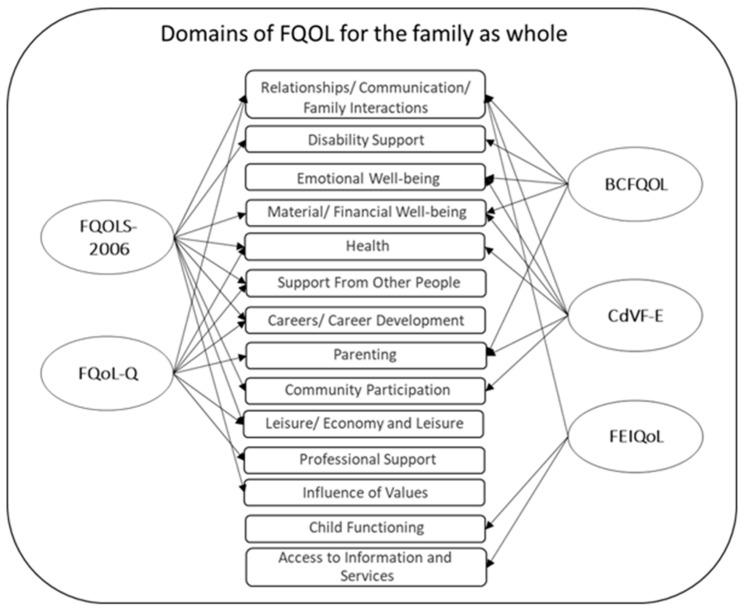
Domains used in the FQoL scales for the family as a whole.

The number of domains used in the current scales ranges from three to nine. Figure 3 displays an overview of all domains used in the five FQoL scales. Relationship, communication, and family interaction are used as the most important predictors of the FQoL in all scales, followed by education, health, and material and financial well-being, which were included in three scales. Most domains are frequently used in at least two scales. The dimensions used in one scale were professional support, influence of values, child functioning, and access to information and services. A significant agreement between *BC-FQoL* and *FQoLS-2006* exists in the domains of well-being, family relationships, and disability-related support. An overview of the respective domains and subdomains covered by the different FQoL scales follows the overview in Figure 3.

**Figure 4:**
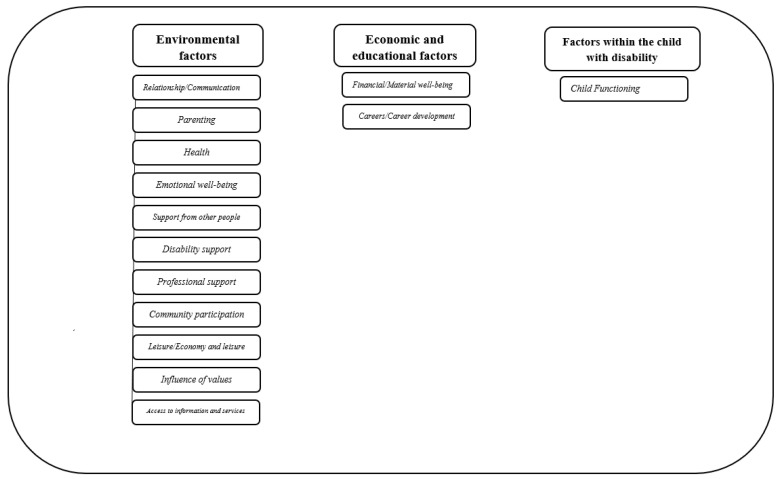
Overarching categories and domains used in the FQoL scales.

Figure 4 shows the domains used in the QoL scales for each family member. The psychological/emotional well-being was included in *WHOQOL-BREF* and *ComQol*, and *ComQol* and *PWI-A* also matched on the three dimensions of health, safety, and community. In contrast, the *QoLA* items were not divided into domains. While the *PWI-A* scale was based on 11 items.

### 3.4. Domains of the FQoL

The domains used in the five FQoL scales were grouped into three overarching categories: environmental factors, economic and educational factors, and factors within the child with disability. This classification helps to clearly structure the multitude of domains included in the scales, which are described in detail below.

### 3.5. Environmental Factors

This overarching category includes all environmental conditions assessed within the five FQoL scales considered in the current study, taking into account both proximal factors from the immediate environment of the child with disabilities (e.g., family, friends) and distal areas of influence (e.g., professional support services, access to information and services). Eleven domains were placed in this category and explained in more detail below.

#### 3.5.1. Relationship/Communication/Family Interactions

The *relationships/community/family interactions* domain encompasses various forms of interpersonal encounters within the family and it is the only category found in all five FQoL scales. Although relationships, communication, and interaction among family members are considered in each of the FQoL scales, several differences can be identified in terms of the subject focus. Accordingly, while the *BC-FQoL* and the *CdVF-E* scale place emphasis on deep, loving relationships between family members, the *FEIQoL* scale strongly concentrates on participation in social activities. While the vast majority of the five scales refer to the current situation, two scales (*FQoLS-2006*, *FQoL-Q*) also contain questions about the future development of the family relationships. In summary, all five scales place great emphasis on the general atmosphere within the family, family connectedness, and perceptions of communication. A detailed description of the items that were used as part of the relationships/communication/family interaction domain can be found below (see Table 2).

#### 3.5.2. Parenting

This domain can be found in three of the five FQoL studies considered, the *BC-FQoL*, the *CdVF-E,* and the *FQoL-Q* scale. Overall, the domain of *parenting* refers to the family’s ability to recognize and meet the child’s individual needs and to provide support in accomplishing everyday tasks (e.g., schoolwork). Both the *BC-FQoL* and *FQoL-Q* scales entail items regarding the family’s ability to promote children’s independence in daily life and the establishment of quality relationships with others. According to the *CdVF-E*, *family adaptation* specifically includes accepting and adjusting to the disability of a loved one [34]. Table 3 provides an overview of the items used in the three different FQoL scales, as part of the *parenting* domain.

#### 3.5.3. Health

In principle, three scales (*CdVF-E*, *FQoLS-2006*, *FQoL-Q*) take into account not only the physical conditions but also the psychological and emotional characteristics of family members. However, the *FQoL-Q* scale focuses specifically on the subjectively perceived well-being over the past week, while the *FQoLS-2006* scale sheds light on the future health development. It is important to emphasize that the *physical and mental health domain* of the *FQoL-Q* corresponds to the two dimensions *of emotional well-being* and *physical/material well-being,* formulated within the *BC-FQoL* scale. Table 4 lists the items and sample items from the respective scales.

#### 3.5.4. Emotional Well-Being

In contrast to *health*, the domain of *emotional well-being* refers exclusively to the emotional state of family members and its maintenance. As can be seen from Table 5, aspects of external support (e.g., friends) that can help promote *emotional well-being* are also mentioned.

#### 3.5.5. Support from Other People

This category encompasses all support measures given by non-service providers (e.g., friends, relatives, neighbors) to families of children with disabilities. Both scales include offers of help related to practical (e.g., shopping, errands), as well as emotional matters (e.g., listening) (see Table 6).

#### 3.5.6. Disability Support

*Disability support* refers to the quality of support provided by professional services to families of children with mental or physical disabilities. Within the two scales dealing with professional disability support, several aspects are considered. Accordingly, the *BC-FQoL* takes into account several different areas of life, namely personal and the vocational/educational domains. It also includes an item that asks about the relationship between family members and service providers. However, the *FQoLS-2006* captures the availability of professional service providers for children with disabilities and the extent to which family members seek assistance. Table 7 provides an overview of the items included in the two scales.

#### 3.5.7. Professional Support

In contrast to *disability support*, *professional support* as formulated in the *FQoL-Q* scale refers to interventions initiated not by individuals but by the nonprofit organizations or by high state institutions, such as the government. Table 8 provides an overview of the items used as part of the *professional support* domain within the *FQoL-Q* scale.

#### 3.5.8. Community Participation

*Community participation* is only included within the framework of two scales, the *CdVF-E* and the *FQoLS-2006* (see Table 9). This category refers to the importance of interactions of children with disabilities and their family members with people and places in their immediate environment. It includes participation in social activities for leisure and recreation. *Community participation* was further developed as part of the *FQoLS-2006* to capture the possible experiences of discrimination [19].

#### 3.5.9. Leisure/Economy and Leisure

According to the *FQoLS-2006*, *Leisure and recreation* focus on “activities for relaxation, entertainment, and fun” [19] (p. 182). This is to express that not only leisure activities but also, for example, political or community involvement, are taken into account if an individual derives pleasure from that activity. The *FQoL-Q* scale, however, combines recreational activities with economic factors. The authors reason that the concept of leisure in China is seen as the opposite of work and employment rather than a form of realization of personal interests, as in the West [35]. Furthermore, as families in China often seek to spend their free time travelling, appropriate financial resources are necessary. Table 10 provides an overview of the items included in the two scales.

#### 3.5.10. Influence of Values

The domain *of influence of values* is found in one scale, exclusively. Accordingly, *influence of values* in the *FQoLS-2006* represents the significance of the impact of various spiritual, cultural, and personal values on people’s daily lives [19]. See Table 11 for a detailed description of the items used, as part of the *FQoLS-2006*.

#### 3.5.11. Access to Information and Services

*Access to information and services* is only considered in the *FEIQoL* scale, which was developed for families which children aged 0–6. It includes the family’s knowledge of their child’s disability, child development, how to manage difficult behaviors, and resources, such as the support services, medical assistance, and organizations in their community. Table 12 lists the items used in the *FEIQoL*.

### 3.6. Economic and Educational Factors

This overarching category refers to all economic and educational/work-related aspects of family life captured in the FQoL scales. Overall, two dimensions (*financial/material well-being* and *careers/career development*) were assigned to this category and described in more detail below.

#### 3.6.1. Financial/Material Well-Being

In contrast to the *FQoLS-2006* and the *CdVF-E* questionnaires, which focus exclusively on economic factors (i.e., financial situation) of the family, *physical/material well-being* as formulated in the *BC-FQoL* scale, also includes aspects related to the health and safety of family members. Overall, all three scales aim to capture family financial resources, as these can often prevent families from achieving a good FQoL [19]. Table 13 provides insights into the items used as part of the three FQoL scales.

#### 3.6.2. Careers/Career Development

*Careers* and *career development* was found in two scales, the *FQoLS-2006* and the *FQoL-Q* (see Table 14). Both scales measure the career satisfaction and success of family members. In addition, the *FQoLS-2006* captures the career preparation and development, and contains an objective indicator, and the employment status of each family member.

### 3.7. Factors within the Child with Disability

Although several scales contain a number of items that consider various factors specifically referring to the individual with the disability (e.g., the *social inclusion and participation* domain in the *CdVF-E* scale or the *disability-related support* domain in the *BC-FQoL* scale), a separate category for child functioning can only be found as part of the *FEIQoL* scale. This seems interesting, as several studies point to the strong influence of child functioning on the overall FQoL e.g., Davis and Gavidia-Payne [51].

#### Child Functioning (Figure 5)

The items used as part of the *FEIQoL* focus on the family’s perception of the child’s health, engagement, independence, and social relationships. It also assesses whether the family is able to support the child financially, involve the child in daily errands, and engage in recreational activities with the child. The small total number of domains considered in the FEIQOL can explain the large amount of items included in the domain *of child functioning*. Table 15 lists the items used in the *FEIQoL* to measure child functioning.

**Figure 5:**
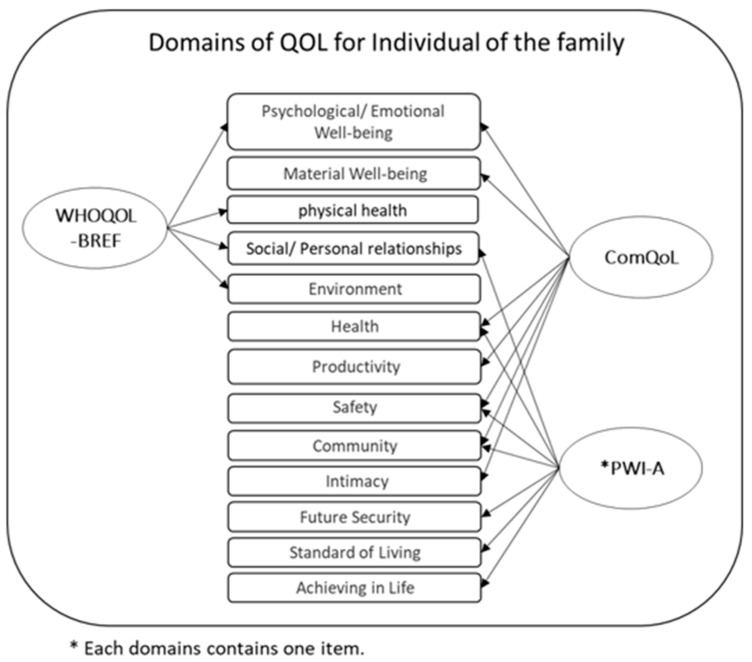
Domains used in the QoL scales for each family member.

### 3.8. Psychometric Properties of the Scales

The scales used to measure the FQoL were validated in terms of their content validity, construct validity, and criterion validity. Content validity was assessed by presentation to experts [52,53,54]. For construct validity, exploratory factor [7,35,55,56,57] and confirmatory factor analyses [35,44,54,56,57,58,59,60,61,62,63,64] were used. Five factors have been identified for the *BC-FQOoL* scale in the CFA in several countries: Turkey [63], Singapore [60], Taiwan [62], Greece [57], Saudi Arabia [58], and Spain. In Greece, the EFA and CFA showed a structure of three factors: family interaction and support, family care, and disability-related support [57]. The stability of the measure confirms that the *BCFQoL* scale is isomorphic for fathers and mothers [65]. In the original English version of the *FEIQoL*, a four-factor structure was found [33], whereas the Spanish version showed that the three-factor structure was preferred after conducting the EFA, and the CFA and was consistent with the theory of Zona and colleagues [56]. Criterion validity was assessed using coincidence validity [40,66,67]. Different types of validity tests were used in some studies, such as, criterion validity [64], convergent and discriminant validity [63], and the Rasch analysis [50].

Reliability was confirmed by the internal consistency and test–retest [36,40,54,56,57,64,68]. In the *FQoLS-2006*, the stability dimension was weakened by the reliability in the nine domains [68] and by the meaning and stability dimension [43].

## 4. Discussion

### 4.1. Conceptualizing the FQoL

The most widely used domain of the scales was family relationships and interactions, which are considered cornerstones for assessing the quality of family life across cultures [18]. Some of the scales were not limited to the closed clauses of the domains (e.g., *FQoLS-2006*), but also asked open-ended questions. These types of scales help provide an overall perception of the FQoL because they use a Likert scale response (e.g., five points) and other observation methods to explain the results [18]. The qualitative data contribute to a more comprehensive understanding and strengthen the findings obtained to explore the perceptions of family satisfaction [34,45].

Regarding the conceptualization of the FQoL, the current review showed that it often includes three key factors: factors within the child with a disability, economic/educational factors as well as environmental factors. The kind of disability the child has been diagnosed with can be identified as the most important predictor of the FQoL, as it is directly linked with many other factors. For instance, the less severe the child’s disability, the less support required from the professionals, the less the parents’ careers are affected, etc. However, the current review also indicated that there are numerous other factors affecting the FQoL. Although most instruments include environmental factors, not all focus on the proximal aspects from the child with disabilities’ immediate environment (e.g., family, friends) and distal spheres of influence (e.g., professional supports). Additionally, economic factors need to be considered for the FQoL. In summary, based on the key theses used to assess the FQoL, the following can be stated: the family quality of life of families, including a person with intellectual disabilities, is based on the functioning of the person with the disability. Further, it is highly influenced by the interplay of the environmental factors (proximal and distal factors), as well as the economic factors. It should be noted, however, that most scales include aspects related to interpersonal, educational, and financial resources. In this context, reference can be made to Pierre Bourdieu’s [69] theory of capital, according to which social, cultural, and economic factors play an important role in promoting human and physical well-being. Interestingly, in the social aspects, the non-family support systems occupy a subordinate place in the scales. This suggests that the initiatives to measure the FQoL, as a whole, need to consider the interpersonal aspects within the family (e.g., relationship, interaction), factors indicative of the educational and occupational opportunities and the satisfaction of family members, and environmental resources.

### 4.2. Scales Assessing the FQoL

This scoping review presented nine scales measuring the QoL of families of children with IDs in 120 studies that met the inclusion criteria. Regarding the FQoL assessment, the review found that the *BC-FQoL* scale is the most commonly used instrument because it is easy to use and includes only 25 items. In addition, it can be used by family services and program providers to obtain information about the needs of families with disabilities [17]. It was also described by some authors as the most well established scale of the FQoL [12,67,70]. In addition to the BC-FQoL, the international *FQoLS-2006*, developed and tested in Australia, Canada, Israel, Taiwan, and South Korea, is the most commonly used scale to measure the FQoL [71].

It turns out that the majority of respondents in the studies were parents, a caregiver, or mothers only, which means that it relies on one or two individuals to assess the FQoL of the family as a whole. When the responses include all family members, this provides robust and validated results for assessing the FQoL [30]. However, as mentioned earlier, the scales in the current review do not ever represent all aspects of the FQoL [6]. This is not possible because none of the scales aim to capture the perspectives of all family members. In addition, studies conducted in different regions of the world could benefit from the use of a specific FQoL scale, as these are designed for use in different countries and thus different cultural environments. Finally, when considering the usefulness of the scales, it may be important to look closely at the purpose of the particular study. For example, research projects aimed at planning and evaluating support for families with children with disabilities could benefit from the use of specific scales (e.g., FQoLS-2006, FQoL-Q). In addition, the QoL scales can be used to examine the impact on the family, of living with a disabled child.

### 4.3. Holistic Approach vs. Individual Approach

Within the review, it was possible to divide the nine scales into two types used in these studies: scales that measure the FQoL for the family as a whole (see Figure 3), and scales that measure the level of satisfaction with the QoL of a particular family member (see Figure 5). Regarding the FQoL scales, the participants’ responses should reflect the satisfaction level of the family as a whole, not just the family member responding to the items. These scales include the *BCFQoL*, *FQoLS-2006*, *CdVF-E*, *FEIQoL*, *FQoL-Q*. However, the other four scales (*WHOQoL-BREF*, *QoLA*, *ComQol*, and *PWI-A*) measure the individual’s QoL and may be useful in some cases, depending on the nature of the planned studies. For example, the QoL scales may be applied if the researcher wants to examine the extent to which the presence of a child with a disability in the family affects certain family members [17]. In this case, it is possible to use the scale with more than one family member and compare the responses to test the validity of this assumption. In summary, both types of scales are important in determining how family members perceive their individual or overall QoL for the family as a whole.

Because most FQoL research comes from developed countries, the samples in the studies reviewed show that certain populations are overrepresented. As a result, it can be seen that research has not yet captured the FQoL from a global perspective, including populations from all over the world [14,15,35]. This highlights the importance of further studies that consider different cultural contexts and verify the psychometric characteristics to provide a reliable basis for implementing programs and plans that improve the QoL of individuals with IDs and their families [54].

### 4.4. Limitations

The current study has some limitations that need to be considered. First, only studies in the English language were considered. Second, limited databases were covered. However, the databases with the most relevant material and widest coverage were selected.

#### Implications for Future Research

Based on the results of our review of more than one hundred studies, it is clear that there are opportunities to improve the field of measuring and understanding the QoL of families of children with disabilities. First, the construct of the FQoL can be further explored through additional theoretical studies. Accordingly, some researchers have questioned whether the existing domains in some scales do not directly represent the families’ quality of life [18]. In addition, it would be important to explore the extent and sources of the FQoL and identify their particular contribution, in order to promote the FQoL. From a practical point of view, the variables that need to be identified are mainly those that have a strong influence and can be (easily) addressed in the context of prevention, intervention, and support.

Second, further research could explore how many perspectives or sources are needed to gain insight into the FQoL. The difficult question of whether a member’s responses represent the QoL level of the family [18], can be explored by examining the correlation between the different members’ responses on an individual scale, related to their level of QoL and the responses on the scales related to the overall QoL perspective. Within this context, it might also be worthwhile reconsidering the concept of the family, as it might be the case that good friends have a stronger influence on happiness than close relatives see e.g., Chopik [72]. From here, it might be useful to work on developing a scale with different versions for different family members and for the member with a disability and to study the relationship between them, in terms of convergence and differentiation.

In addition, the representativeness of the existing research can be improved by combining studies with samples from different populations that differ culturally and linguistically, to test whether the hypothesized construct of the scale holds across different populations. Furthermore, there are opportunities for studies that use more advanced statistical techniques to examine the properties of the items, such as the item response theory approach or the Rasch analysis, and to examine the psychometric properties of these scales. Lastly, it is important that the FQoL measure encompasses multiple domains, as well as qualitative and quantitative components [6].

## Figures and Tables

**Table 1:** FQoL scales.

Scales	Number of Uses	Domains	Items	Response Options	Stages of Development of the Scale/Psychometric Properties
The Beach Center Family Quality of Life (BCFQoL) [24]	55	Five: family interactions, parenting, emotional well-being, physical/material well-being, and disability-related support.	25	Five-point Likert	Step-1: literature reviews, focus groups, and individual interviews. Step-2: three rounds of EFA+CFA. (from 112 items down to the final 25 items)
The Family Quality of Life Survey (FQoLS-2006) [25]	28	Nine: health, financial well-being, family relationships, support from other people, support from services, influence of values, careers, leisure, and community interaction. six dimensions: attainment, satisfaction, importance, opportunities, initiative and stability.	54	Five-point Likert	Step-1: meetings and discussions with stakeholders. Step-2: In two rounds, the field test was conducted in five countries + CFA for the sub-scales. A short version has been developed and experimentally tested and validated by the face. (from 121 items down to the final 54 items)
Families in Early Intervention Quality of Life (FEIQoL) [33]	3	Three: family relationships, access to information and services, and child functioning	40	Five-point Likert	Step-1: [32] English version was prepared with reference to scales, and some items were prepared by the authors. Step-2: translated into Spanish with four factors. Step 3: EFA+ CFA (from four factors down to the final three factors, 40 items)
Family quality of life for families with a member with an ID, under and over 18 years old (CdVF-E) [34]	6	Seven: emotional well-being, financial well-being, family interaction, organization and parenting, family adjustment, health, and social inclusion and participation.	61: CdVF-E < 18 67: CdVF-E > 18	Five-point Likert	Step-1: focus groups with families of people with intellectual disabilities. Step-2: identify the preliminary elements of the survey and review them by experts. Step-3: apply to a trial sample. Step-4: field test. (from 105 items down to the final 61 items on the under 18 scale, and 67 items on the over 18 scale)
Family Quality of Life Questionnaire Chinese (FQoL-Q) [35]	1	Seven: economy and leisure, physical and mental health, parenting, family communication, support from others, professional support, and career development.	35	Five-point Likert	Step-1: building the scale, based on 1. Zona et al.’s theory; 2. the three most important scales for the FQoL; 3. culture and challenges for Chinese families. Step-2: EFA + CFA. (from 72 items down to the final 35 items)
WHO Quality of life (BREF WHOQOL-BREF) [36]	18	Four: physical health, psychological well-being, social relationships, and environment	26	Five-point Likert	Step-1: an international group of researchers to place cross-culturally appropriate fields in 15 international centers across 12 languages. Step-2: apply the different samples and select the top 100 named (WHOQOL-100) items and identify them in six domains. Step-3: create the short version (WHOQOL-BREF) with four domains and 26 items (discriminant validity, CFA, internal consistency, and test–retest reliability).
Quality of Life in Autism Questionnaire (QoLA) [37]	1	Consisting of two sub-scales: the first (QoL subscale) includes 28 items designed to measure the parents’ overall perception of their quality of life;the second measures the impact of ASD symptoms subscale.	28	Five-point Likert	Step-1: counseling, semi-structured interviews, and review of the many general measures of the quality of life used and the diagnostic criteria for the autism spectrum disorder. Step-2: construct validity: independent sample t-tests were performed to compare the total scores between the clinical and control groups+ concurrent and convergent validity.
Personal Wellbeing Index (PWI) [38]	1	Seven: standard of living, health, achievement in life, personal relationships, safety, community involvement, and future security.	7	Eleven-point Likert	Step-1: created from the Comprehensive Quality of Life scale, the domains were identified through a literature review. Step-2: re-developed by a group of researchers from several countries and tested in the field Step-3: construct validity, convergent validity, reliability(In addition: modify the wording of some items, and add an additional optional field spiritual/religious)
Comprehensive Quality of Life Scale (ComQoL [39]	1	Seven: material well-being, health, productivity, intimacy, safety, place in community, and emotional well-being.		Five-point Likert	

**Table 2:** Items/item examples considered in the domain of relationships/communication/family interactions within each FQoL scale.

FQoL Scale	Domain Name	Items/Item Examples
**BC-FQoL:** The Beach Center Family Quality of Life [24]	Family interaction	My family enjoys spending time together.
		Family members talk openly with each other.
		My family solves problems together.
		My family members support each other to accomplish goals.
		My family members show that they love and care for each other.
		My family is able to handle life’s ups and downs.
**CdVF-E:** Family quality of life for families with a member with an ID under and over 18 years old [34]	Family climate	All my family members show love and affection towards each other.
		All the members of my family, including brothers and sisters and close relatives, try to create a pleasant family environment.
**FQoLS-2006:** The Family Quality of Life survey [25]	Family relationships	How important are your family relationships to your family’s quality of life?
		Are there opportunities for members of your family to maintain and enhance good relationships with each other?
		Do members of your family make efforts to keep good relationships within your family?
		To what degree do members of your family enjoy good relationships with each other?
		In the near future, is it likely that your family relationships will …? If improve or decline, why?
		All things considered, how satisfied are you with the relationships within your family?
		Please provide any additional information or explanations that you would like.
**FEIQoL:** Families in Early Intervention Quality of Life [33]	Family relationships	Our family time is spent with our child.
		Our family time is spent one-on-one with each of our children.
		The communication within our family.
		Our family’s ability to solve problems together.
**FQoL-Q:** Family Quality of Life questionnaire Chinese [35]	Family communication	My family members help each other.
		My family members respect each other’s hobbiesand personal space.
		My family members will fight together for the future of our family.
		I’m happy with the relationships in my family.
		My family members trust each other.

**Table 3:** Items/item examples considered in the domain of parenting within each FQoL scale.

FQol Scale	Domain Name	Items/Item Examples
**BC-FQoL:** The Beach Center Family Quality of Life [24]	Parenting	Family members help the children to be independent.
		Family members help the children with schoolwork and activities.
		Family members teach the children how to get along with others.
		Adults in my family teach the children to make good decisions.
		Adults in my family know other people in their children’s lives (i.e., friends, teachers).
		Adults in my family have time to take care of the individual needs of every child.
**CdVF-E:** Family quality of life for families with a member with an ID under and over 18 years old [34]	Family adaptation	My family adapts to the needs of the relative with the IDD.
**FQoL-Q:** Family Quality of Life questionnaire Chinese [35]	Parenting	My family develops skills to prepare children for life in the future.
		My family focuses on thinking about children’s future.
		My family helps children learn to be independent.
		My family teaches children how to get along with others.
		My family helps children finish schoolwork.

**Table 4:** Items/item examples considered in the domain of health within each FQoL scale.

FQoL Scale	Domain Name	Items/Item Examples
**CdVF-E:** Family quality of life for families with a member with an ID under and over 18 years old [34]	Health	My family member with an ID has healthy eating habits.
**FQoLS-2006:** The Family Quality of Life survey [25]	Health	How important is your family’s health to your family’s quality of life?
		Are there opportunities in your area for your family’s health needs to be met?
		Do members of your family make efforts to maintain or improve their health, such as engaging in regular exercise, paying attention to diet?
		To what degree do members of your family enjoy goodhealth?
		In the near future, is it likely that your family’s current level of health will …? If improve or decline, why?
		All things considered, how satisfied are you with the health of your family?
**FQoL-Q: **Family Quality of Life questionnaire Chinese [35]	Physical and mental health	For nearly a week, my family members have been in good health without any discomfort.
		For nearly a week, my family has been emotionally stable.
		For nearly a week, my family has been upbeat about life.
		For nearly a week, my family has been feeling safe.
		For nearly a week, my family members have been sleeping very well.
		For nearly a week, my family’s appetite has been very good.

**Table 5:** Items/item examples considered in the domain of emotional well-being within each FQoL scale.

FQoL Scale	Domain Name	Items/Item Examples
**BC-FQoL:** The Beach Center Family Quality of Life [24]	Emotional well-being	My family has the support we need to relieve stress.
		My family members have friends and others who provide support.
		My family members have some time to pursue their own interests.
		My family has outside help available to us that take care of the special needs of family members.
**CdVF-E:** Family quality of life for families with a member with an ID under and over 18 years old [34]	Emotional well-being	My family is hopeful and has projects for the future.

**Table 6:** Items considered in the domain of support from other people within each FQoL scale.

FQoL Scale	Domain Name	Items
**FQoLS-2006:** The Family Quality of Life Survey [25]	Support from other people	How important to your family’s quality of life is the practical and emotional support you receive from other people, excluding service providers?
		Are there opportunities to receive practical and emotional support from other people, excluding service providers, should your family need it?
		Do members of your family make the effort to receive practical and emotional support from other people, excluding service providers?
		To what degree does your family receive practical and emotional support from other people, excluding service providers?
		In the near future, is it likely that the practical and emotional support you receive from other people, excluding service providers, will …? If improve or decline, why?
		All things considered, how satisfied are you with the practical and emotional support your family receives from other people, excluding service providers?
**FQoL-Q:** Family Quality of Life questionnaire Chinese [35]	Support from others	Relatives help with my family’s daily routine, such as shopping and taking care of the family.
		Relatives provide emotional support for my family, such as encouragement and listening.
		Neighbors help with my family’s routine, such as shopping or taking care of the family.
		Friends help with my family’s routine, such as shopping or taking care of the family.
		Friends provide emotional support for my family, such as encouragement and listening.

**Table 7:** Items considered in the domain of disability support within each FQoL scale.

FQoL Scale	Domain Name	Items
**BC-FQoL:** The Beach Center Family Quality of Life [24]	Disability-related support	My family member with special needs has the support to make progress at school or in the workplace.
		My family member with special needs has the support to make progress at home.
		My family member with special needs has the support to make friends.
		My family has good relationships with the service providers who work with our family member with a disability.
**FQoLS-2006:** The Family Quality of Life Survey [25]	Support from services	How important to your family’s quality of life is the support from the intellectual or developmental disability related services?
		Are there opportunities in your area to receive the intellectual or developmental disability related services your family needs?
		Do members of your family make the effort to obtain the disability related services they need?
		To what degree are your family’s needs, related to the family member(s) with an intellectual or developmental disability, being met by the services in your area?
		In the near future, is it likely that the support your family receives from the disability related services will …? If improve or decline, why?
		All things considered, how satisfied are you with the disability related services your family receives?

**Table 8:** Items considered in the domain of professional support within each FQoL scale.

FQoL Scale	Domain Name	Items
**FQoL-Q:** Family Quality of Life questionnaire Chinese [35]	Professional support	My family can receive social support from foundations, nonprofit organizations, volunteers, and others.
		My family can receive financial support from the government (e.g., the civil affairs bureau and disabled persons’ federation) for their children.
		My family can receive the relevant medical and rehabilitation support from the government (such as the civil affairs bureau and the disabled persons’ federation).
		I am satisfied with the professional support services my family receives.

**Table 9:** Items/item examples considered in the domain of community participation within each FQoL scale.

FQoL Scale	Domain Name	Items/Item Examples
**CdVF-E:** Family quality of life for families with a member with an ID under and over 18 years old [34]	Social inclusion and participation	The family member with an ID has a group of friends.
**FQoLS-2006:** The Family Quality of Life survey [25]	Community interaction	How important to your family’s quality of life is it for members of your family to interact with people and places in your community?
		Are there opportunities for members of your family to interact with people and places in your community?
		Do members of your family make the effort to interact with people and places in your community?
		To what degree does your family make the effort to interact with people and places in your community?
		In the near future, is it likely that your family’s interaction with people and places in your community will …? If improve or decline, why?
		All things considered, how satisfied are you with your family’s interaction with people and places in your community?

**Table 10:** Items considered in the domain of leisure/economy and leisure within each FQoL scale.

FQoL Scale	Domain Name	Items
**FQoLS-2006: **The Family Quality of Life survey [25]	Leisure and recreation	How important are leisure and recreation to your family’s quality of life?
		Are there opportunities for your family members to engage in leisure and recreation activities?
		Do members of your family make the effort to take part in leisure and recreation activities?
		To what degree do your family members engage in leisure and recreation activities?
		In the near future, is it likely that your family’s leisure and recreation will …? If improve or decline, why?
		All things considered, how satisfied are you with your family’s leisure and recreation?
**FQoL-Q:** Family Quality of Life Questionnaire Chinese [35]	Economy and leisure	All family members can participate in leisure activities.
		My family will actively engage in leisure activities.
		My family has sufficient opportunities to participate in leisure activities.
		I am satisfied with how relaxed my family members are.
		My family can make ends meet.
		My family members have convenient transportation tools to travel where they want to go.
		My family has a suitable family environment.

**Table 11:** Items considered in the domain of influence of values within each FQoL scale.

FQoL Scale	Domain Name	Items
**FQoLS-2006:** The Family Quality of Life survey [25]	Influence of values	How important to your family’s quality of life are personal, spiritual, religious and/or cultural values?
		Are there opportunities for your family members to develop and hold personal, spiritual, religious and/or cultural values that can contribute to your family’s quality of life?
		Do members of your family make the effort to maintain or strengthen personal, spiritual, religious and/or cultural values that contribute to your family’s quality of life?
		To what degree do your family members hold personal, spiritual, religious and/or cultural values that contribute to your family’s quality of life?
		In the near future, is it likely that the personal, spiritual, religious and/or cultural values that contribute to your family’s quality of life will…? If improve or decline, why?
		All things considered, how satisfied are you with the degree to which personal, spiritual, religious and/or cultural values contribute to your family’s quality of life?

**Table 12:** Items considered in the domain of access to information and services within each respective FQoL scale.

FQoL Scale	Domain Name	Items
**FEIQoL:** Families in Early Intervention Quality of Life [33]	Access to information and services	Our family’s connections and organizations about our child’s special need.
		Our family’s access to health care.
		The information our family has about our child’s condition or disability.
		The information our family has about resources, including services.
		The information our family has about what to do with our child.
		Our family’s knowledge about how children in a similar situation to ours learn.
		The information our family has about child development.
		Our family’s access to services for our child.
		Our family’s knowledge on what to do when our child engages in difficult behavior.
		Our family’s knowledge about parenting.

**Table 13:** Items/item examples considered in the domain of financial/material well-being within each FQoL scale.

FQoL Scale	Domain Name	Items/Item Examples
**BC-FQoL:** The Beach Center Family Quality of Life [24]	Physical/material well-being	My family receives medical care when needed.
		My family receives dental care when needed.
		My family members have transportation to travel to the places they need to be.
		My family has a way to take care of our expenses.
		My family feels safe, at home, work, school, and in our neighborhood.
**FQoLS-2006:** The Family Quality of Life survey [25]	Financial well-being	How important is financial well-being to your family’s quality of life?
		Are there opportunities for members in your family to earn enough money to do the things your family wants?
		Do members of your family make the effort to maintain or improve the financial situation of your family?
		To what degree does your family’s financial situation meet your family’s expectations?
		In the near future, is it likely that your family’s financial situation will …? If improve or decline, why?
		All things considered, how satisfied are you with the financial well-being of your family?
**CdVF-E:** Family quality of life for families with a member with an ID under and over 18 years old [34]	Financial well-being	My family can cover the cost of basic needs (food, clothing, etc.).

**Table 14:** Items considered in the domain of careers/career development within each FQoL scale.

FQoL Scale	Domain Name	Items
**FQoLS-2006:** The Family Quality of Life survey [25]	Careers and preparing for careers	How important is it to your family’s quality of life, for family members to pursue or prepare for the careers they want?
		Are there opportunities for members of your family to pursue the careers they want and attend the schools they want?
		Do members of your family make the effort to develop their education and/or careers?
		To what degree have your family members been able to prepare for and have the education and careers they want?
		In the near future, is it likely that your family’s ability to pursue and prepare for the careers they want will …? If improve or decline, why?
		All things considered, how satisfied are you with your family’s careers and ability to prepare for those careers?
**FQoL-Q:** Family Quality of Life questionnaire Chinese [35]	Career development	My family will pursue work or study that they love.
		My family is doing well at work.
		My family members are satisfied with their current jobs.

**Table 15:** Items considered in the domain of child functioning within each FQoL scale.

FQoL Scale	Domain Name	Items
**FEIQoL:** Families in Early Intervention Quality of Life [33]	Child functioning	Our child’s ability to fall asleep and stay asleep.
		Our child’s health.
		Our family’s ability to take our child on routine errands (grocery store, mall, haircut, dentist, doctor, etc.).
		Our child’s ability to get along with his/her brother/sister(s).
		How welcomed we feel in our faith-based community.
		Our child’s independence.
		Our family’s ability to take our child on social outings (movies, zoo, library).
		Our child’s participation in home and community routines.
		Our family’s ability to pay for things.
		Our child is getting along with other children.
		Our child is participating in school or group care activities.
		Our child is expressing him- or herself.
		Our child is playing with toys and using objects.
		Our child is understanding what is said to him or her.
		Our child is getting along with adults.
		Our child is behaving appropriately.
		Thinking about your child’s overall life situation now, would you describe it as
		The support available to our family to help our child make friends.

## Data Availability

Not Applicable.
